# Noninvasive Intracranial Pressure Prediction Using a Multimodal Ultrasound-Based Hemispheric Modeling Strategy: A Prospective Dual-Center Study

**DOI:** 10.1007/s12028-025-02339-5

**Published:** 2025-08-11

**Authors:** Jun Qiu, Tong-Juan Zou, Dong-Mei Wang, Hai-Rong Luo, Hai-Tao Yu, Ling Lei, Wan-Hong Yin

**Affiliations:** 1 Department of Critical Care Medicine, West China Hospital of Sichuan University, Chengdu, Sichuan China; 2 Department of Critical Care Medicine, The Second People’s Hospital of Yibin Yibin, Sichuan, China; 3 Department of Critical Care Medicine, Xindu District People’s Hospital, Chengdu, Sichuan China

**Keywords:** Intracranial pressure, Multimodal ultrasound, Hemispheric modeling, Optic nerve sheath diameter, Transcranial doppler

## Abstract

**Background:**

Accurate intracranial pressure (ICP) surveillance is a cornerstone of neurocritical care management, yet invasive monitoring still depends on neurosurgical expertise, specialized hardware, and continuous bedside resources—factors that restrict universal use even though insertion-related complications are relatively uncommon. Contemporary noninvasive ultrasound methods have limited predictive accuracy and seldom incorporate affected side information. We therefore preliminarily validated a multimodal, ultrasound-based hemispheric modeling approach that blends hemodynamic and structural indexes while emphasizing affected side specificity to enhance ICP prediction.

**Methods:**

In this prospective, dual-center study, 41 neurosurgical patients provided 216 paired ultrasound and invasive ICP measurements. Affected side and contralateral ultrasound parameters including pulsatility index, resistance index, optic nerve sheath diameter (ONSD), optic disk height, and ONSD-to-eyeball diameter ratio were analyzed. Linear mixed-effects models (LMMs) predicted continuous ICP, whereas generalized LMMs classified elevated ICP (≥ 20 mm Hg).

**Results:**

Affected side parameters showed consistently stronger ICP correlations than unaffected side parameters. An affected side five-parameter LMM (pulsatility index, resistance index, ONSD, ONSD-to-eyeball diameter ratio, and optic disk height) provided superior continuous ICP prediction (coefficient of determination [*R*^2^] = 0.618, root mean square error = 0.424), significantly outperforming contralateral models (*R*^2^ = 0.338, root mean square error = 0.558). For binary classification, affected side ONSD demonstrated excellent accuracy (area under the receiver operating characteristic curve = 0.927, sensitivity = 91.4%, specificity = 79.5%), whereas the optimal affected side seven-parameter generalized LMM reached an area under the receiver operating characteristic curve of 0.829 (sensitivity = 80.8%, specificity = 75.8%).

**Conclusions:**

This study demonstrated the feasibility and potential advantages of a novel hemispheric (side-specific) modeling strategy for noninvasive ICP assessment. The multiparameter model constructed using affected side ultrasound parameters exhibited promising predictive accuracy, providing a potentially valuable and innovative noninvasive approach for ICP monitoring in neurocritical care patients that may serve as an adjunct when invasive monitoring is unavailable, although further validation is warranted.

## Introduction

Intracranial pressure (ICP) monitoring is fundamental in the treatment of patients with severe neurologic injuries such as traumatic brain injury, intracerebral hemorrhage, and brain tumors [1, 2]. When ICP remains persistently above 20 mm Hg, cerebral perfusion pressure may fall, leading to cerebral ischemia, edema, and herniation—events that are rapidly fatal if untreated [3]. The current gold standard for measuring ICP is invasive monitoring, most commonly with intraventricular catheters or intraparenchymal probes [4]. These devices are widely employed in neurocritical care (approximately 59% intraparenchymal and 36% ventricular in one large study) [4]. Although major complications such as infection or hemorrhage are relatively uncommon [5–7], the need for specialized expertise and dedicated equipment still limits universal implementation [4].

In recent years, noninvasive ICP monitoring techniques have gained increasing attention. Ultrasonographic methods, in particular, have emerged as powerful tools for ICP estimation due to their convenience, real-time capability, and noninvasiveness [8, 9]. For example, transcranial color Doppler (TCCD) ultrasound can indirectly reflect changes in ICP by measuring hemodynamic parameters of the middle cerebral artery (MCA) such as pulsatility index (Pi) and resistance index (Ri) [10]. Studies have shown that as ICP rises, Pi and Ri increase while end-diastolic velocity (Vd) and mean flow velocity (Vm) decrease [11]. However, TCCD measurements can be influenced by systemic factors such as blood pressure and arterial carbon dioxide levels, which may limit their accuracy [12]. Pi is generally more sensitive to ICP changes than Ri [11], but we included both indexes to comprehensively assess hemodynamic changes.

Optic nerve sheath diameter (ONSD) is another important ultrasound-derived parameter that can be measured noninvasively via transorbital sonography [13]. Because the optic nerve sheath is continuous with the intracranial subarachnoid space, elevated ICP is transmitted to the sheath, causing ONSD expansion [14]. Numerous studies have found a strong positive correlation between ONSD and ICP, and an enlarged ONSD provides high sensitivity and specificity for detecting ICP ≥ 20 mm Hg [15, 16]. Compared with TCCD, ONSD is less affected by systemic hemodynamic fluctuations, but it does have limitations such as operator dependence and variability due to individual anatomy [17].

Recent investigations have proposed novel ultrasound indexes to improve ICP estimation. Notably, optic disk height (ODH) and the ONSD-to-eyeball transverse diameter ratio (ONSD/ETD) have been explored as additional markers [18, 19]. ODH is an ultrasound measure of papilledema (optic disk swelling), whereas the ONSD/ETD ratio normalizes the sheath diameter to eyeball size, helping to mitigate interindividual anatomical differences and potentially improve the stability of ICP prediction [20]. Nonetheless, the accuracy of any single ultrasound parameter for predicting ICP remains limited, highlighting the need for a multiparameter combined assessment [21].

Although several groups have combined multiple ultrasound parameters to enhance ICP prediction [22, 23], they have rarely distinguished measurements from the affected side versus the contralateral (healthy) hemisphere, leaving the effect of focal pathology (e.g., mass effect or edema) underexplored. Moreover, prior work often relied on conventional statistics that did not account for the correlation inherent in repeated observations from the same patient [24]. Here, we address these gaps by adopting a side-specific (“hemispheric”) design that compares the predictive performance of affected side and contralateral ultrasound metrics for noninvasive ICP estimation.

## Methods

### Study Design and Participants

This prospective study was conducted at two tertiary-care centers—West China Hospital of Sichuan University (Chengdu, Sichuan, China) and The Second People’s Hospital of Yibin (Yibin, Sichuan, China)—after approval by their institutional review boards. For presentation and modeling, the centers are labeled as Site A and Site B in tables and figures (West China Hospital of Sichuan University = Site A; The Second People’s Hospital of Yibin = Site B). Adults aged 15–75 years requiring invasive ICP monitoring for clinical management were eligible. The inclusion criteria were as follows: age 15–75 years; neurosurgical patients requiring invasive ICP monitoring (for conditions such as traumatic brain injury, intracerebral hemorrhage, or brain tumor); and ability to undergo ultrasound examinations with adequate image quality. The exclusion criteria were as follows: history of severe ocular or optic nerve disorders, hyperostosis or absence of an acoustic window (temporal bone window) preventing effective TCCD measurement, severe ocular trauma upon admission or during hospitalization, severe failure of major organs (heart, lung, kidney) prior to surgery, hemodynamic shock, withdrawal from the study, or loss to follow-up. The data exclusion criteria were as follows: ultrasound image quality was poor; the recorded ICP value was grossly inconsistent with the patient’s clinical condition; and ultrasound parameter measurements yielded outlier values that deviated unreasonably from physiological norms. For example, if an invasive ICP reading was artifactually high (such as during flushing of an external ventricular drain) without corresponding clinical signs, that measurement was excluded. Similarly, extreme ultrasound values beyond physiological plausibility (e.g., an abnormally large ONSD due to a poor acoustic window) were discarded to ensure data quality.

Written informed consent was obtained from each patient or their legal representative before inclusion. For patients unable to provide consent (e.g., those in coma), consent was obtained from their legally authorized representatives. All procedures were performed in accordance with the Declaration of Helsinki and relevant regulations.

### Data Collection

All ultrasound examinations were performed with a Mindray M9 portable color Doppler system (2–4-MHz phased-array probe for transcranial Doppler [TCD]; 7–12-MHz linear probe for ocular imaging). For every patient, we obtained at least two paired ICP ultrasound measurements per day, usually once in the morning (at approximately 08:00–10:00) and once in the afternoon (at approximately 14:00–16:00), both chosen when the patient was hemodynamically and neurologically stable. If the clinical picture suggested a sudden change in ICP (e.g., acute neurological deterioration), extra measurements were added. During each session, the patient laid in a supine resting position; for those with an external ventricular drain the drain was clamped for several minutes before and throughout the measurement to ensure an accurate ICP reading.

**Fig. 1:**
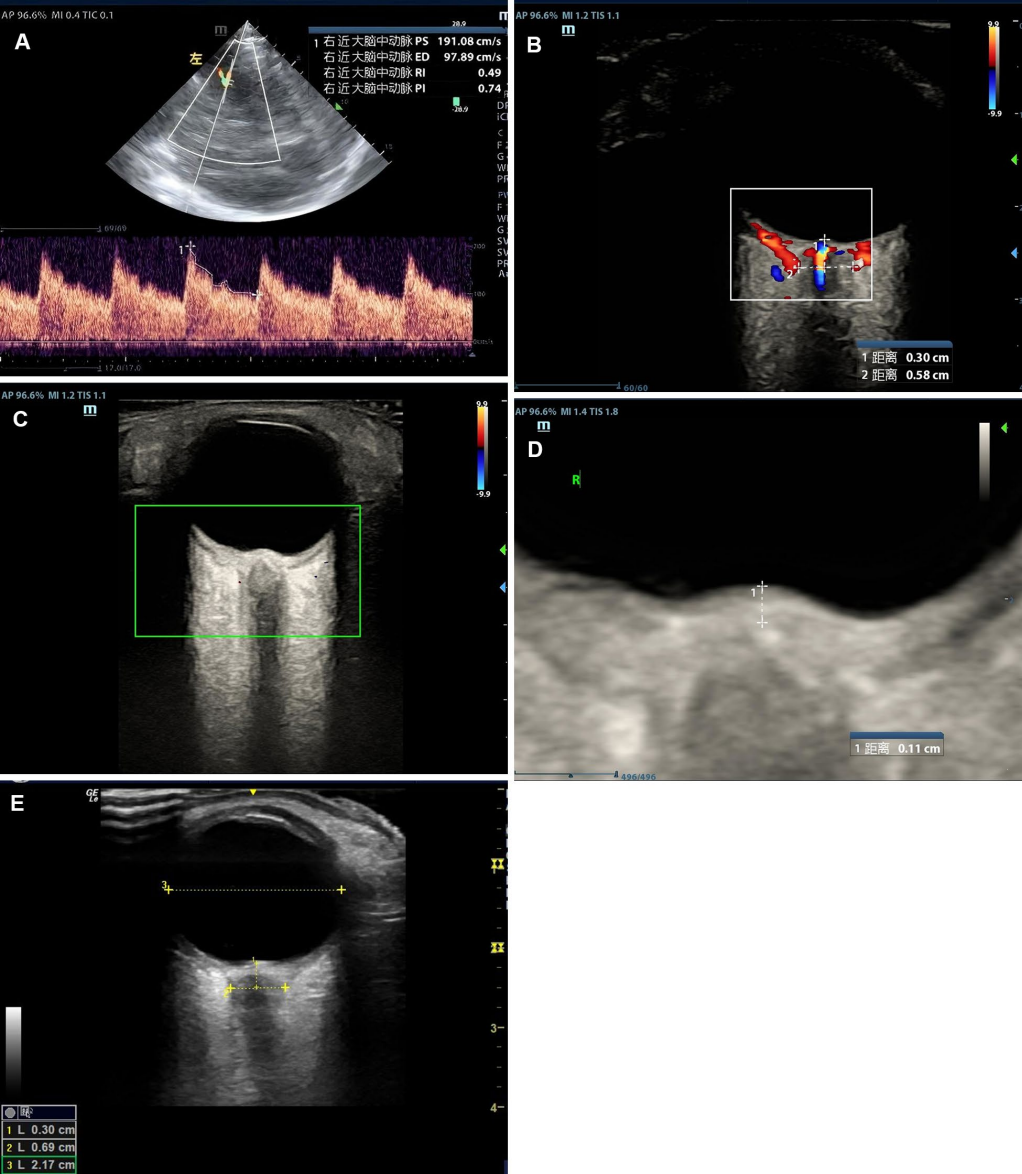
Representative ultrasound images from patients with different intracranial pressure (ICP) levels. (**A**) A patient with aneurysmal subarachnoid hemorrhage (ICP = 17 mmHg): transcranial color Doppler (TCCD) via the temporal window shows an end-diastolic flow velocity (Vd) of 97.9 cm/s, mean flow velocity (Vm) of 128.9 cm/s, resistance index (Ri) = 0.49, and pulsatility index (Pi) = 0.74. (**B**) A patient with ICP = 22 mmHg: orbital ultrasound reveals an optic nerve sheath diameter (ONSD) of 5.8 mm measured 3 mm behind the optic disc. (**C**) A patient with ICP = 45 mmHg: marked papilledema is observed, indicating elevated optic disc height (ODH). (**D**) Magnified view of the optic disc in (C), with ODH measured at 1.1 mm. (**E**) A patient with ICP = 34 mmHg: ONSD = 0.69 cm and eyeball transverse diameter = 2.17 cm, yielding an ONSD/ETD ratio of 0.32. All images were obtained with written informed consent from the patients

TCCD of the MCA was obtained through the temporal window, recording Pi, Ri, Vd, and Vm. Ocular ultrasound was performed with a linear probe placed gently on the closed upper eyelid. Following a standardized procedure adapted from the Color Doppler–Low power examination–Optic disk clarity–Safety (short examination duration)–Elevate frequency–Dual measurements (CLOSED) protocol [25], color-Doppler mode was activated to highlight the central retinal artery and the surrounding optic-nerve sheath, enhancing sheath-border definition and minimizing artifact. ONSD was measured 3 mm posterior to the optic disk; three measurements were taken for each eye and averaged to improve reliability. In the same view, ODH and ETD were recorded, and the ONSD/ETD was calculated. Representative images of these parameters across different ICP levels are shown in Fig. 1.

To minimize physiological variability and improve the reliability of ICP and ultrasound measurements, all patients were treated under a standardized sedation and ventilation protocol. Sedation was achieved with continuous intravenous infusion of propofol or midazolam, combined with opioids such as fentanyl, remifentanil, or sufentanil, as clinically appropriate (targeting a deep level of sedation, e.g., Richmond Agitation-Sedation Scale of − 4 to − 5, applied similarly at both centers). Patients were maintained in normothermia throughout, as fever or hypothermia can alter cerebral blood flow and Doppler indexes [26, 27]. Intracranial hypertension was managed per standard neurocritical care guidelines (in line with contemporary traumatic brain injury management protocols) [28], including head elevation, cerebrospinal fluid drainage (for external ventricular drains [EVDs]), hyper-osmolar therapy, and adjustment of ventilation parameters, as needed. Other aspects of critical care (hemodynamic and respiratory support, infection management, etc.) were provided according to institutional protocols.

### Statistical Analysis

All statistical analyses were performed using SPSS (v26.0, IBM Corp) and R software (v4.2.2, R Foundation). Continuous variables were expressed as means ± standard deviations or medians with interquartile ranges (IQRs), and categorical variables were expressed as counts with percentages. Group comparisons were made using Student’s *t*-test or Mann–Whitney *U*-test for continuous variables and the *χ*^2^ test or Fisher’s exact test for categorical variables, as appropriate. A two-tailed *P* < 0.05 was considered statistically significant.

We employed linear mixed-effects models (LMMs) to model continuous ICP and generalized LMMs (GLMMs) (with a logit link) for binary ICP elevation status (ICP ≥ 20 mm Hg). These mixed-effects models account for both fixed effects (population-average effects of ultrasound variables) and random effects (study participant–specific deviations), thus appropriately handling the nonindependence of repeated measurements within the same patient. Random intercepts for patient identification were included in all models to capture study participant–level baseline differences. We first built univariable LMMs for each ultrasound parameter to assess individual predictive performance. We then used least absolute shrinkage and selection operator (Lasso) regression for variable selection in a multivariable context, identifying key predictors from the full set of candidates. The optimal Lasso model (determined via cross-validation) informed the choice of variables for the final multiparameter LMM. We compared three multivariable LMMs constructed using (1) affected side parameters, (2) contralateral-side parameters, and (3) bilateral mean parameters. Model goodness-of-fit was evaluated using the marginal *R*^2^ (variance explained by fixed effects) and root mean square error (RMSE).

For classification of ICP ≥ 20 mm Hg, we similarly evaluated both single-parameter GLMMs and multiparameter GLMMs. Area under the receiver operating characteristic curve (AUC), sensitivity, specificity, and accuracy were calculated for each model. We used DeLong’s test to compare AUCs. All model coefficient estimates are presented with 95% confidence intervals.

Measurement consistency and interrater reliability for ultrasound measures were assessed using Bland–Altman plots and the intraclass correlation coefficient (ICC). A two-tailed *P* value < 0.05 was considered statistically significant for all tests.

## Results

### Study Population Characteristics

A total of 41 patients yielded 216 paired ICP ultrasound measurements. Baseline characteristics are detailed in Table 1. The mean age was 49.73 ± 16.78 years (range 15–74), and 25 patients (61.0%) were male. Site A enrolled 24 patients (58.5%) and site B enrolled 17 patients (41.5%), with no significant age-related or sex-related differences between sites (*P* > 0.5). The affected hemisphere was right in 24 patients (58.5%) and left in 17 patients (41.5%). Etiologically, traumatic brain injury predominated (20 patients, 48.8%), followed by intracerebral hemorrhage (14, 34.1%) and other neurological disorders (e.g., brain tumors; 7, 17.1%). Neurological severity at admission was high: the median Glasgow Coma Scale (GCS) was 6 (IQR 3–8); 31 patients (75.6%) were classified as severe (GCS ≤ 8), 6 (14.6%) as moderate (GCS 9–12) and 4 (9.8%) as mild (GCS ≥ 13). Ten patients (24.4%) had undergone primary decompressive craniectomy before intensive care unit (ICU) admission; this subgroup was too small for separate analysis, and their ultrasound-ICP patterns followed the overall cohort trend. All patients underwent invasive ICP monitoring: approximately 75.6% received intraparenchymal probes and 24.4% received EVDs. The median monitoring duration was 5 days (IQR 3–7 days).

**Table 1:** Baseline characteristics of the study participants (*N* = 41)

Category	Subcategory	Value
Age (years)	Mean ± SD	49.73 ± 16.78
Sex	Male	25 (61.0%)
	Female	16 (39.0%)
Hospital	Site A	24 (58.5%)
	Site B	17 (41.5%)
Intracranial Pathology	Traumatic brain injury	20 (48.8%)
	Intracerebral hemorrhage	14 (34.1%)
	Other neurological disorders	7 (17.1%)
Affected Side	Right	24 (58.5%)
	Left	17 (41.5%)
GCS on admission	Median [IQR]	6 [3-8]
	Severe (GCS ≤ 8)	31 (75.6%)
	Moderate (GCS 9–12)	6 (14.6%)
	Mild (GCS ≥ 13)	4 (9.8%)
ICP Monitoring	Intraparenchymal probe	31 (75.6%)
	External ventricular drain	10 (24.4%)

Baseline ultrasound parameters and ICP values are presented in Table 2. Median ICP across measurements was 16.0 mm Hg (IQR 10.0–22.0). The mean Pi and Ri were higher on the affected versus contralateral side (Pi: 1.18 ± 0.44 vs. 1.08 ± 0.43; Ri: 0.64 ± 0.12 vs. 0.61 ± 0.12, respectively). Vms were slightly lower on the affected side, consistent with increased downstream resistance. Mean ONSD was greater on the affected side (5.50 ± 0.72 mm vs. 5.30 ± 0.68 mm), as was median ODH (0.50 mm vs. 0.30 mm). ONSD/ETDs were similar bilaterally (~ 0.25 ± 0.04). Bilateral averages typically fell between affected and contralateral values.

**Table 2:** Baseline ultrasonographic parameters and ICP by side (lesion side, contralateral side, and overall average)

Parameter	Lesion Side (Affected)	Contralateral Side (Unaffected)	Bilateral Average
Intracranial pressure (ICP) (mmHg)	–	–	16.00 [10.00, 22.00]
Pulsatility index (pi)	1.18 ± 0.44	1.08 ± 0.43	1.13 ± 0.42
Resistance index (Ri)	0.64 ± 0.12	0.61 ± 0.12	0.62 ± 0.12
End-diastolic velocity (Vd) (cm/s)	34.78 [26.51, 40.88]	38.58 [30.10, 46.95]	36.28 [28.63, 43.65]
Mean velocity (Vm) (cm/s)	46.45 ± 17.51	49.30 ± 16.76	47.88 ± 16.49
Optic nerve sheath diameter (ONSD) (mm)	5.50 ± 0.72	5.30 [4.65, 5.80]	5.41 ± 0.73
Optic disk height (ODH) (mm)	0.50 [0.05, 0.60]	0.30 [0.00, 0.60]	0.38 (0.10, 0.60)
ONSD/ETD ratio	0.25 ± 0.04	0.24 ± 0.04	0.25 ± 0.04

Values are presented as mean ± SD or median [IQR] as appropriate

### Correlations Between ICP and Ultrasound Parameters

**Fig. 2:**
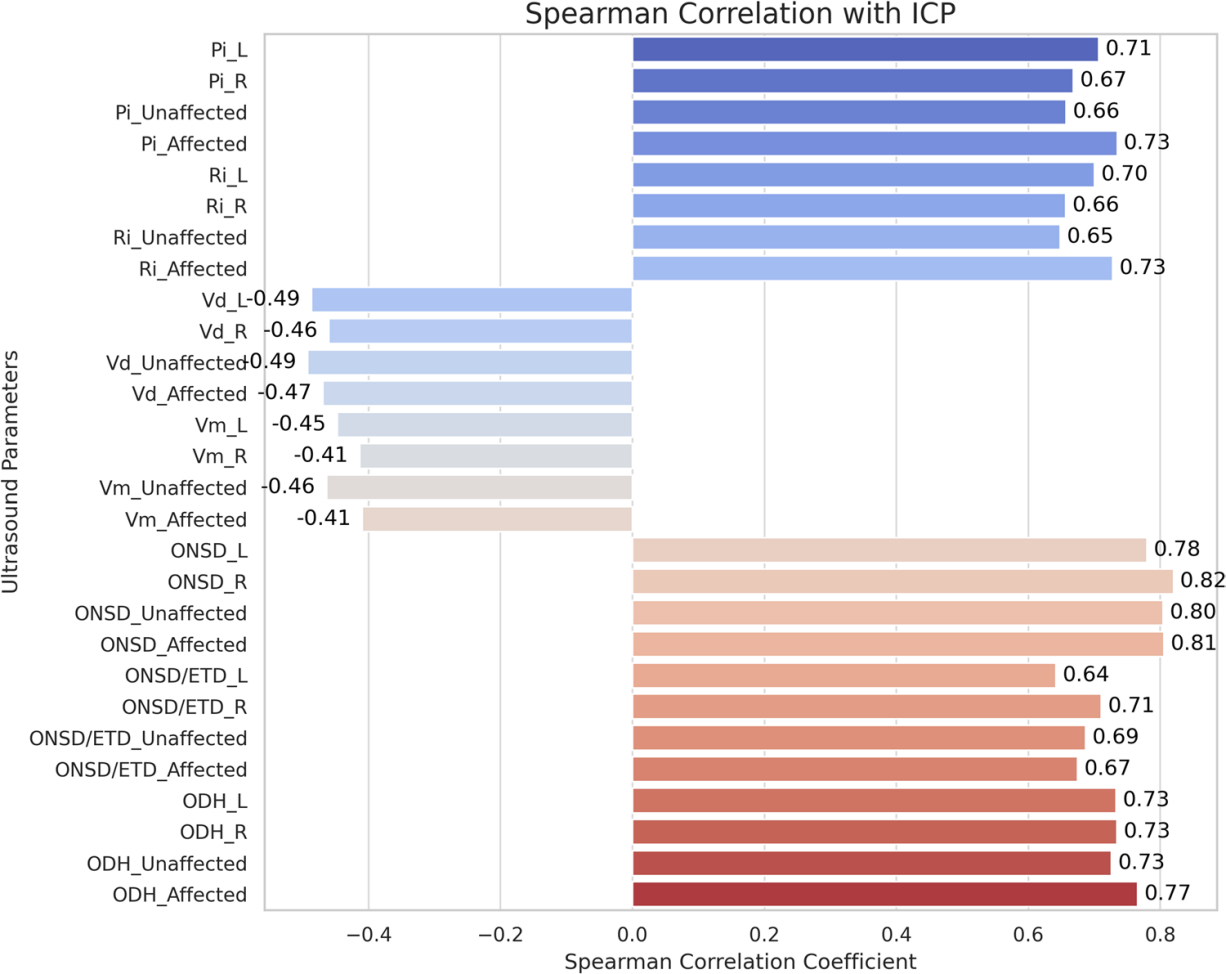
Spearman correlations between ICP and ultrasonographic parameters. Ultrasound-derived parameters from the affected and unaffected sides showed generally strong correlations with invasively measured ICP. ONSD_Affected (r = 0.805), Pi_Affected (r = 0.735), and ODH_Affected (r = 0.765) were among the stronger associations, suggesting that lesion-side parameters may offer higher sensitivity for noninvasive ICP assessment

Correlation analyses revealed significant associations between ICP and multiple ultrasound parameters (Table 3, Fig. 2). ICP correlated strongly and positively with affected side Pi (*r* = 0.735), Ri (*r* = 0.728), ONSD (*r* = 0.805), ODH (*r* = 0.765), and ONSD/ETD (*r* = 0.675; all *P* < 0.001). Conversely, significant negative correlations were observed with affected side Vd (*r* = − 0.469) and Vm (*r* = − 0.409; both *P* < 0.001). Correlations on the affected side were consistently stronger than those on the contralateral side (Table 3).

**Table 3:** Spearman correlation coefficients (r) between ICP and ultrasound parameters measured from lesion (affected) side, contralateral (unaffected) side, and bilateral mean values

Ultrasound Parameter	Lesion side (affected) r	Contralateral side (unaffected) r	Bilateral mean (average) r	*P* value
Pulsatility index (pi)	0.735	0.658	0.724	< 0.001
Resistance index (Ri)	0.728	0.649	0.711	< 0.001
End-diastolic velocity (Vd)	-0.469	-0.492	-0.51	< 0.001
Mean velocity (Vm)	-0.409	-0.463	-0.47	< 0.001
Optic nerve sheath diameter (ONSD)	0.805	0.804	0.818	< 0.001
ONSD/Eyeball Diameter ratio (ONSD_ETD)	0.675	0.688	0.696	< 0.001
Optic disk height (ODH)	0.765	0.726	0.775	< 0.001

All correlations presented are statistically significant (*P* < 0.001). Positive r-values indicate positive correlations with ICP, while negative r-values indicate inverse correlations

**Fig. 3:**
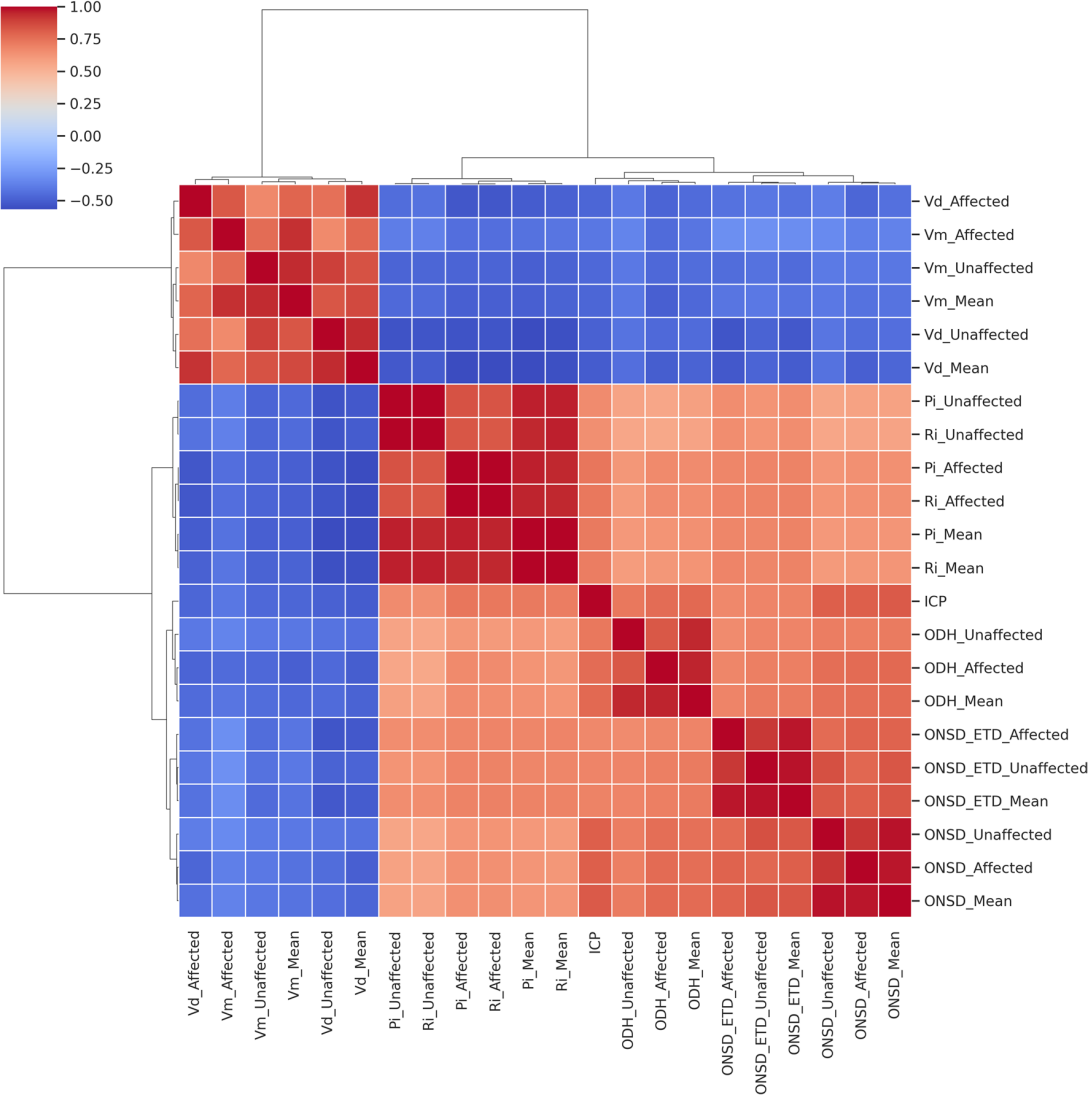
Hierarchical clustering heatmap of ultrasound parameters correlated with ICP. This heatmap classified the parameters into two groups: (1) Vd and Vm-related parameters, which showed negative correlations with ICP; and (2) Pi, Ri, ONSD, ONSD/ETD, and ODH-related parameters, which showed positive correlations with ICP. There were strong positive inter-correlations among the second group of parameters, especially among measurements taken on the lesion side

Hierarchical clustering analysis further identified two distinct parameter groups (Fig. 3): a velocity-based cluster (Vd, Vm) negatively associated with ICP, and a pulsatility/optic nerve cluster (Pi, Ri, ONSD, ONSD/ETD, ODH) positively associated with ICP. Parameters within the second cluster, especially on the affected side, exhibited notably strong intercorrelations.

### Feature Selection and Variable Importance

**Fig. 4:**
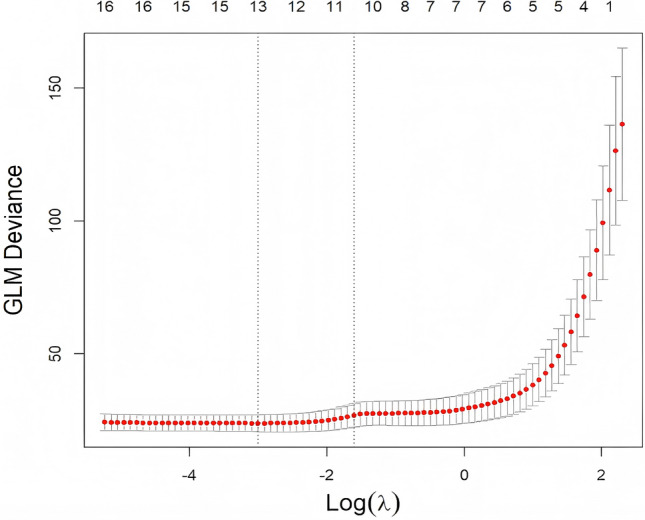
Cross-validation results for Lasso regression to determine the optimal penalty parameter (λ). The horizontal axis represents log(λ), and the vertical axis shows the binomial deviance. Red dots indicate the mean cross-validation error for each λ, with error bars representing the standard error. Two vertical dashed lines mark: (1) the λ value that minimizes the deviance (left), and (2) the largest λ within one standard error of the minimum (right), corresponding to the most parsimonious model

To assess the relative importance of each candidate variable for ICP prediction, we performed Lasso regression analysis. Using cross-validation, the optimal penalty parameter was determined to be λ = 0.028, at which point the Lasso model tended to retain affected side variables preferentially, suggesting their greater predictive value (Fig. 4). For example, in the Lasso model, the affected side Pi, affected side Ri, and affected side ODH had regression coefficients of 26.44, − 59.24, and 2.54, respectively, indicating these parameters contributed substantially to explaining variance in ICP.

### Continuous ICP Prediction Models

#### Univariable Model Performance

We first evaluated the performance of individual ultrasound parameters in predicting the ICP value using separate univariable LMMs. Table 4 summarizes the results for each parameter. In general, affected side parameters exhibited better predictive performance (higher *R*^2^and lower error) than their counterparts from the contralateral side. Among all predictors, the Pi provided the best single-parameter prediction for ICP: the model using the bilateral mean Pi achieved the highest *R*^2^(0.867) and lowest RMSE (0.364), followed closely by the model using affected side Pi (*R*^2^ = 0.864, RMSE = 0.368) and the model using contralateral Pi (*R*^2^ = 0.837, RMSE = 0.403). Hemodynamic indexes such as Pi and Ri were markedly more predictive of ICP than flow velocity measures such as Vd and Vm (Table 4).

**Table 4:** Performance of univariable LMM models for ICP prediction (sorted by descending *R*^2^)

Variable	MSE	RMSE	MAE	*R*^2^
Pi_Mean	0.132	0.364	0.257	0.867
Pi_Affected	0.135	0.368	0.263	0.864
Pi_Unaffected	0.162	0.403	0.284	0.837
Ri_Mean	0.177	0.421	0.288	0.822
ONSD_Mean	0.178	0.422	0.277	0.821
ONSD_ETD_Mean	0.181	0.425	0.293	0.818
Ri_Affected	0.187	0.433	0.3	0.812
ONSD_ETD_Affected	0.197	0.444	0.306	0.802
ONSD_Affected	0.202	0.449	0.299	0.797
ONSD_ETD_Unaffected	0.211	0.459	0.308	0.788
Ri_Unaffected	0.215	0.464	0.317	0.784
ONSD_Unaffected	0.215	0.464	0.298	0.784
ODH_Mean	0.223	0.472	0.326	0.776
ODH_Unaffected	0.251	0.501	0.354	0.748
ODH_Affected	0.272	0.521	0.36	0.727
Vd_Unaffected	0.4	0.633	0.38	0.598
Vd_Mean	0.406	0.637	0.374	0.592
Vd_Affected	0.427	0.654	0.39	0.571
Vm_Unaffected	0.429	0.655	0.401	0.569
Vm_Mean	0.43	0.656	0.392	0.568
Vm_Affected	0.448	0.669	0.403	0.55

#### Multivariable Model Performance

We next constructed multiparameter LMM models incorporating all ultrasound variables selected by Lasso and compared models based on different hemispheric data. The model that included all seven affected side parameters achieved *R*^2^ = 0.558 and RMSE = 0.456, substantially better than the corresponding model using seven contralateral-side parameters (*R*^2^ = 0.338, RMSE = 0.558) and the model using seven bilateral mean parameters (*R*^2^ = 0.448, RMSE = 0.510). Within the affected side full model, the strongest contributing predictors were affected side Pi (*β* = 1.229, *P* < 0.001), affected side Ri (*β* = − 0.686, *P* < 0.001), and affected side ODH (*β* = 0.321, *P* = 0.001).

**Fig. 5:**
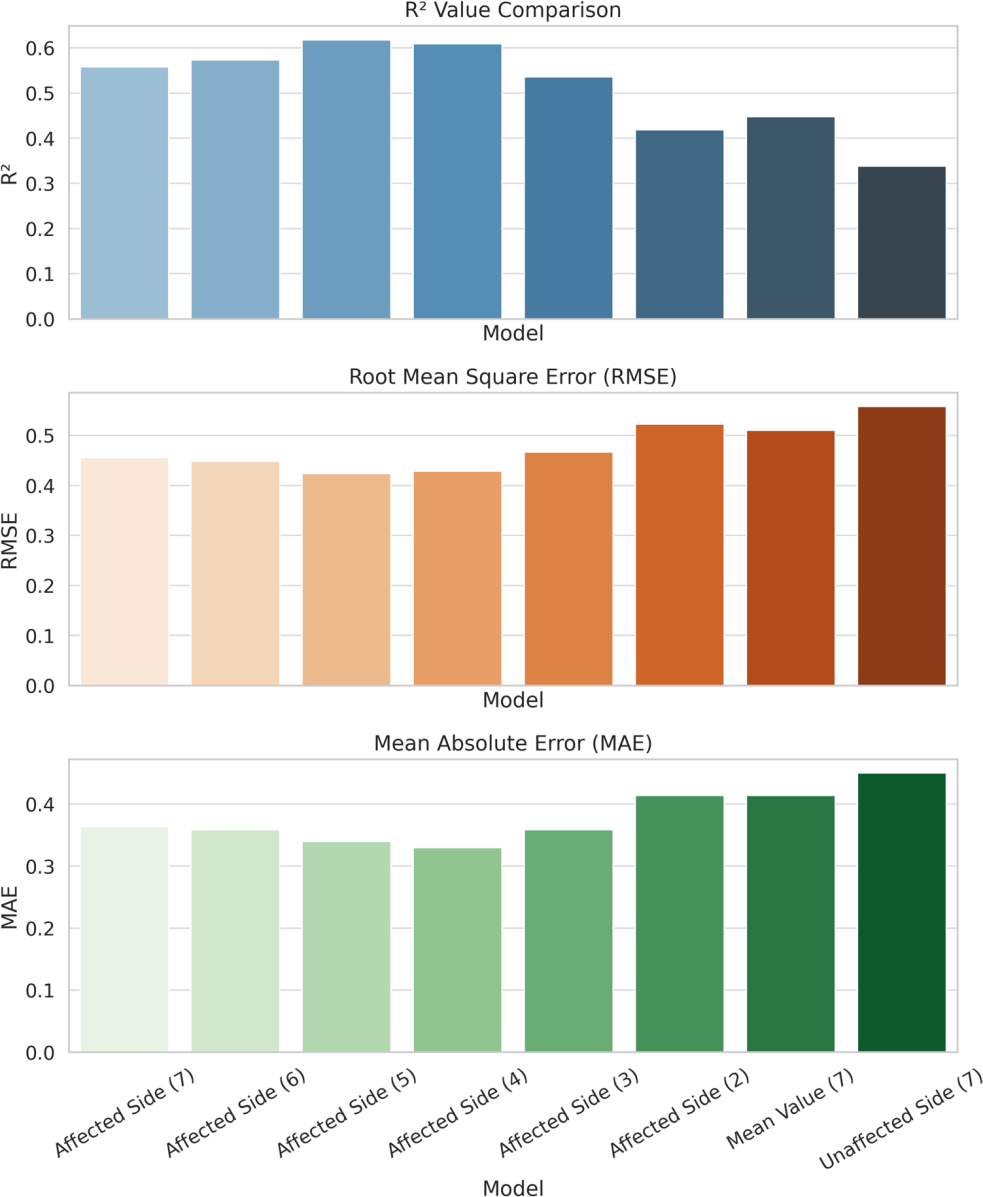
Comparison of multivariable LMM model performance by side. This bar graph illustrates the R², RMSE, and MAE of the multi-parameter models. The five-parameter model using lesion-side variables had the best performance (highest R² and lowest RMSE/MAE). Models based on bilateral mean or contralateral-side parameters showed considerably lower predictive accuracy

To avoid overfitting and improve practicality, we then optimized the affected side model by reducing the number of input variables. Table 5 and Fig. 5 present the performance of affected side models with sequentially reduced feature sets, compared against models using contralateral or averaged parameters. Notably, the highest model fit was achieved by an LMM that retained five key affected side parameters (Pi, Ri, ONSD, ONSD/ETD, and ODH), which yielded *R*^2^ = 0.618 and RMSE = 0.424. This five-variable affected side model outperformed models that included a larger number of parameters, indicating that adding additional ultrasound variables beyond these five did not improve accuracy. The fixed-effects equation of the optimal five-parameter LMM was:

$$\begin{aligned} {\text{nICP }} = \, & 0.0{15} + { 1}.{212} \times {\text{Pi}}\_{\text{Affected}} - 0.{656} \times {\text{Ri}}\_{\text{Affected}} + \, 0.{136} \times {\text{ONSD}}/{\text{ETD}}\_{\text{Affecte}} \\ & \quad - 0.0{34} \times {\text{ONSD}}\_{\text{Affected }} + 0.{319} \times {\text{ODH}}\_{\text{Affected}}. \, \\ \end{aligned}$$

**Table 5:** Comparison of multiparameter LMM model performance using lesion side vs. contralateral vs. averaged parameters

Model Type	Number of Parameters	R^2^	RMSE	MAE
Affected Side – 7 parameters	7	0.558	0.456	0.363
Affected Side – 6 parameters	6	0.573	0.448	0.359
Affected Side – 5 parameters (optimal)	5	0.618	0.424	0.340
Affected Side – 4 parameters	4	0.609	0.429	0.330
Affected Side – 3 parameters	3	0.536	0.467	0.359
Affected Side – 2 parameters	2	0.419	0.523	0.414
Unaffected Side – 7 parameters	7	0.338	0.558	0.450
Bilateral Mean – 7 parameters	7	0.448	0.510	0.414

In which nICP denotes the predicted noninvasive ICP in mm Hg; the constants are the model intercept and coefficients derived from the LMM’s fixed effects.

#### Binary Classification of ICP Elevation

We further evaluated the ability of various models to classify whether ICP was elevated above the clinical threshold of 20 mm Hg. The diagnostic performance of each single-parameter GLMM and selected multiparameter GLMM combinations is summarized in Tables 6 and 7.

**Table 6:** Diagnostic performance of single-parameter GLMM models for elevated ICP (ICP ≥ 20 mmHg)

Parameter	Source	AUC	Cutoff	Sensitivity	Specificity	PPV	NPV	Accuracy
Pi	Affected	0.915	1.235	82.9%	91.8%	82.9%	91.8%	82.9%
Pi	Unaffected	0.872	1.155	75.7%	85.6%	71.6%	88.0%	71.6%
Pi	Mean	0.906	1.152	85.7%	84.9%	73.2%	92.5%	73.2%
Ri	Affected	0.914	0.677	82.9%	91.1%	81.7%	91.7%	81.7%
Ri	Unaffected	0.867	0.653	75.7%	85.6%	71.6%	88.0%	71.6%
Ri	Mean	0.899	0.650	82.9%	84.2%	71.6%	91.1%	71.6%
Vd	Affected	0.770	28.935	67.1%	87.0%	71.2%	84.7%	71.2%
Vd	Unaffected	0.779	34.435	68.6%	78.8%	60.8%	83.9%	60.8%
Vd	Mean	0.792	31.792	68.6%	84.2%	67.6%	84.8%	67.6%
Vm	Affected	0.737	40.040	61.4%	83.6%	64.2%	81.9%	64.2%
Vm	Unaffected	0.757	44.885	75.7%	73.3%	57.6%	86.3%	57.6%
Vm	Mean	0.765	40.265	61.4%	85.6%	67.2%	82.2%	67.2%
ONSD	Affected	0.927	5.550	91.4%	79.5%	68.1%	95.1%	68.1%
ONSD	Unaffected	0.931	5.650	82.9%	91.8%	82.9%	91.8%	82.9%
ONSD	Mean	0.936	5.575	91.4%	84.9%	74.4%	95.4%	74.4%
ONSD/ETD	Affected	0.898	0.255	81.4%	81.5%	67.9%	90.2%	67.9%
ONSD/ETD	Unaffected	0.899	0.245	80.0%	82.9%	69.1%	89.6%	69.1%
ONSD/ETD	Mean	0.907	0.252	80.0%	85.6%	72.7%	89.9%	72.7%
ODH	Affected	0.888	0.550	75.7%	86.3%	72.6%	88.1%	72.6%
ODH	Unaffected	0.856	0.450	74.3%	82.9%	67.5%	87.1%	67.5%
ODH	Mean	0.890	0.525	75.7%	88.4%	75.7%	88.4%	75.7%

**Table 7:** Diagnostic performance of multivariable GLMM models for predicting ICP ≥ 20 mmHg

Model Type	Number of parameters	AUC	P value	Sensitivity	Specificity	PPV	NPV
Affected Side – 7 parameters	7	0.829	0.002	80.8%	75.8%	56.8%	90.9%
Unaffected Side – 7 parameters	7	0.806	< 0.001	80.8%	74.2%	55.3%	90.7%
Bilateral Mean – 7 parameters	7	0.787	1	76.9%	80.3%	60.6%	89.8%
Affected Side – 6 parameters	6	0.826	0.004	80.8%	75.8%	56.8%	90.9%
Affected Side – 5 parameters	5	0.814	0.009	80.8%	75.8%	56.8%	90.9%
Affected Side – 4 parameters	4	0.817	0.485	69.2%	86.4%	66.7%	87.7%
Affected Side – 3 parameters	3	0.802	0.906	61.5%	90.9%	72.7%	85.7%
Affected Side – 2 parameters	2	0.801	0.009	80.8%	72.7%	53.8%	90.6%

AUC, area under ROC curve; PPV, positive predictive value; NPV, negative predictive value. *P*-values indicate significance of the model (comparison of AUC to 0.5)

In the single-parameter GLMM models (Table 6), ONSD was the most effective individual predictor of ICP elevation. The affected side ONSD model achieved an AUC of 0.927, with 91.4% sensitivity and 79.5% specificity for detecting ICP ≥ 20 mm Hg. Contralateral-side ONSD and the bilateral mean ONSD performed similarly (AUC 0.931 and 0.936, respectively). Following ONSD, the next best single predictors were Pi and Ri (AUCs generally in the 0.87–0.92 range, depending on side), whereas Vd and Vm had comparatively lower AUCs around 0.77–0.79. These results indicate that optic nerve sheath measurements provide the highest diagnostic accuracy among individual ultrasound metrics for identifying intracranial hypertension.

**Fig. 6:**
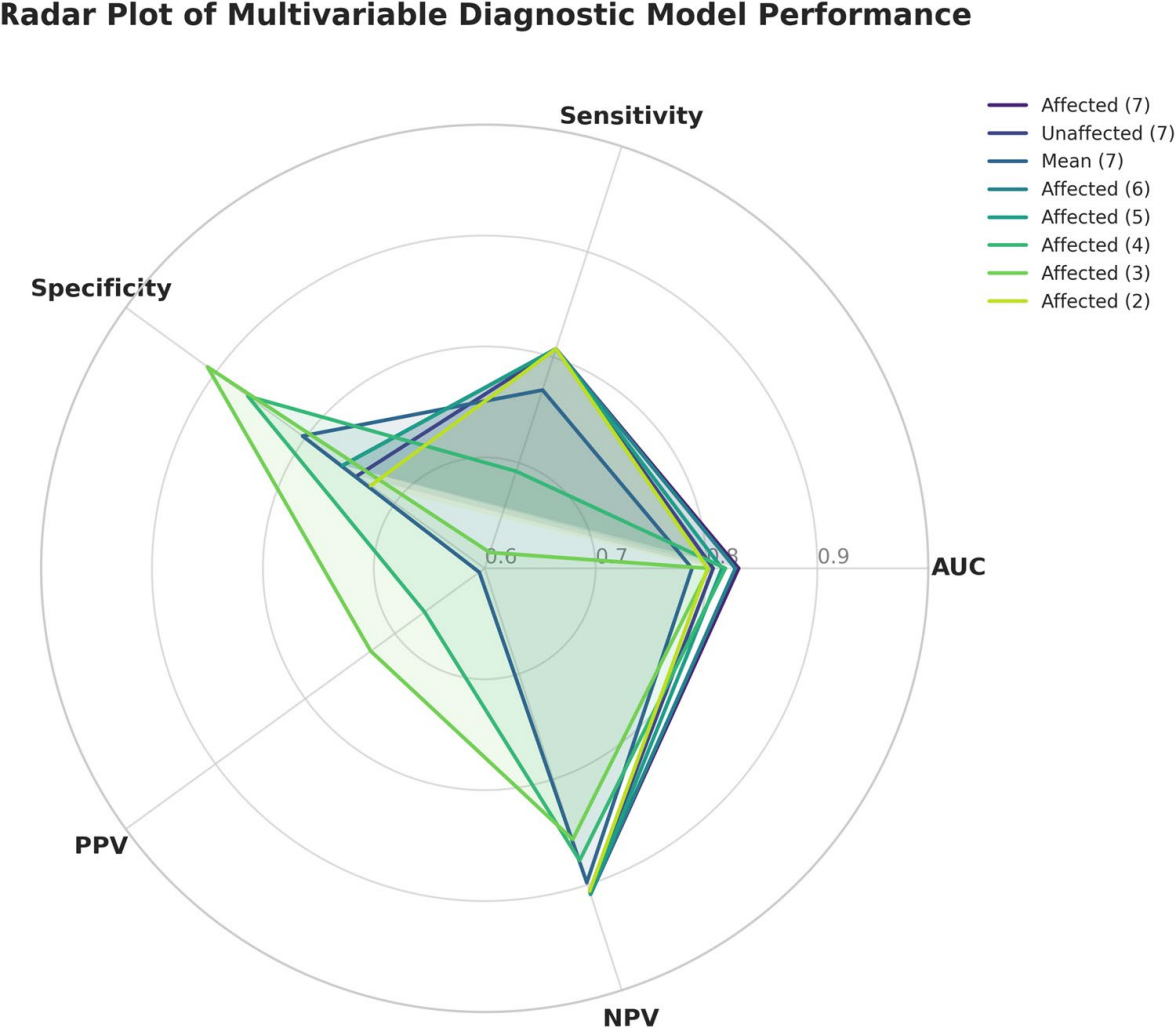
Radar chart of diagnostic performance for various multi-parameter GLMM models. The radar chart plots five diagnostic metrics (AUC, sensitivity, specificity, PPV, NPV) for eight different GLMM models. The sevenparameter lesion-side model shows the best overall performance across all metrics compared to models using contralateral or averaged parameters, highlighting its superiority in detecting ICP elevation

In multiparameter GLMM models for ICP ≥ 20 mm Hg (Table 7), the model combining all seven affected side variables yielded the highest AUC (0.829) among the multivariable models, slightly better than the seven-parameter models based on contralateral side data (AUC 0.806) or on bilateral averages (AUC 0.787). We then incrementally reduced the affected side model complexity to examine whether a simpler combination could perform as well. Notably, the full seven-parameter affected side model consistently showed the best overall performance across metrics; removing variables tended to decrease the AUC. Figure 6 presents a radar chart of five diagnostic metrics (AUC, sensitivity, specificity, positive predictive value, and negative predictive value) for several GLMM models, illustrating that the seven-parameter affected side model achieved the most balanced and superior performance profile. In practical terms, this suggests that incorporating all available ultrasound parameters from the affected side yields the most accurate classification of ICP status in our cohort.

#### Measurement Consistency and Reliability Assessment

**Fig. 7:**
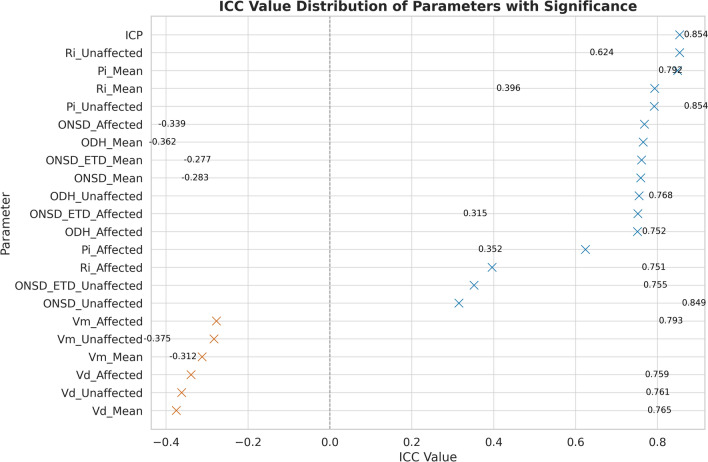
Intraclass correlation coefficients (ICC) for repeated ultrasound parameter measurements. This figure shows the ICC values for repeated measurements of each ultrasound-derived parameter. Parameters with positive ICCs (blue markers) indicate greater between-subject than within-subject variability, reflecting good measurement reliability. Parameters with negative ICCs (orange markers) reflect higher within-subject variability, suggesting less consistent measurements. Notably, ICP (ICC = 0.854), ONSD_Affected (0.768), and ODH_Affected (0.751) demonstrated high repeatability. All ICC values shown were statistically significant (P 0.001)

To evaluate the reproducibility and reliability of the measurements, Bland–Altman plots and ICC were used (Fig. 7). Most core parameters demonstrated moderate-to-high measurement consistency, particularly ICP (ICC = 0.854), affected side ONSD (ICC = 0.768), and affected side ODH (ICC = 0.751), indicating robust stability across repeated measurements. Although certain parameters, such as Vd and Vm, exhibited comparatively lower ICC values, likely reflecting physiological variability associated with transient blood flow conditions, this did not substantially affect the overall credibility of the models. Collectively, these findings suggest that the key modeling variables in this study have high measurement reliability and that the dataset maintains an acceptable consistency for reliable analyses.

## Discussion

Continuous ICP monitoring is fundamental in neurocritical care, yet current noninvasive approaches have not achieved the reliability needed to replace invasive methods. In this dual-center study, we developed and prospectively evaluated a novel “hemispheric” noninvasive ICP monitoring strategy that emphasizes side-specific ultrasound measurements. Our results indicate that an affected side–oriented strategy integrating multiple ultrasound modalities yields more accurate and more stable ICP estimates than conventional approaches that rely on a single parameter or overlook hemispheric asymmetry.

### Clinical Significance of Hemispheric Modeling

We found that ultrasound parameters measured on the affected side (affected hemisphere) bore stronger relationships with invasively measured ICP than those from the contralateral side. This aligns with the intuitive understanding that pathological processes (e.g., focal edema, hematoma, or tumor) in one hemisphere can locally elevate ICP or alter compliance, leading to greater perturbations in adjacent ultrasound measurements (TCD flow dynamics and ONSD). By explicitly separating measurements by side, our hemispheric modeling approach captured these asymmetries. Importantly, the affected side multiparameter model outperformed models based on contralateral or bilaterally averaged data. This highlights that when it comes to noninvasive ICP estimation, where one measures (relative to affected location) can be as crucial as what one measures. Incorporating side specificity allowed us to harness the most informative signals (from the affected side) rather than diluting them with contralateral data that may be relatively less perturbed.

### Value of the Multiparameter Approach

No single ultrasound metric is a perfect surrogate for ICP. Consistent with prior studies [21], we observed only moderate performance when using individual parameters in isolation. For example, ONSD alone was highly sensitive for detecting elevated ICP in our cohort (AUC ~ 0.93 for ICP ≥ 20 mm Hg), in agreement with numerous reports supporting the role of ONSD as a screening tool [15, 16]. TCCD-derived Pi or Ri alone also showed good but slightly lower diagnostic performance. Our multiparameter LMM, however, provided a more robust continuous ICP prediction (*R*^2^ ~ 0.62 for the best affected side model) than any single-parameter model. By combining complementary indicators—hemodynamic (Pi, Ri) and structural (ONSD, ODH, etc.)—the model could account for multiple facets of how raised ICP manifests ultrasonographically. Interestingly, the bilateral mean Pi was the strongest single predictor of ICP among all individual features. This underscores the influence of global cerebral hemodynamics on ICP: averaging Pi from both sides likely captures an overall elevation in downstream cerebrovascular resistance associated with ICP rises. Nevertheless, relying solely on bilateral Pi could overlook lateralized pathology. Our affected side multiparameter model achieved similar predictive accuracy while also incorporating focal information from the affected side and other modalities (such as ONSD). Thus, despite the strength of bilateral Pi as an indicator, a combined approach offers a more comprehensive and stable assessment of ICP. Moreover, by using an LMM framework, we implicitly calibrated for patient-specific differences (via random effects), which further enhanced predictive performance by accounting for individual baseline variability.

### Application of LMM for Repeated Measures

In neurocritical care research, it is common for each patient to have multiple ICP measurements over time, which introduces intrapatient correlation and violates the independence assumption of ordinary least-squares regression. Our use of LMMs is a notable methodological improvement: as noted by Zeiler et al. [24], ignoring the clustered nature of such data can underestimate standard errors and inflate false positives. By including patient-level random intercepts (and random slopes when appropriate), the LMM controlled for within-patient correlations, thereby improving the reliability of our statistical inferences [29]. In our study, using an LMM not only increased prediction accuracy but also enabled us to capture heterogeneity in how different patients’ ICP responded to changes in ultrasound parameters (through examination of random effects), potentially providing a basis for individualized monitoring.

### Interpretation of Model Variables

The Lasso variable selection highlighted affected side Pi, Ri, and ODH as particularly important contributors in the multivariable context. The prominence of Pi is unsurprising—it has long been recognized as an index that rises with intracranial compliance loss and increased ICP. Ri similarly rises, even though, in our final LMM, its coefficient was negative after accounting for Pi. This counterintuitive finding likely results from multicollinearity: Pi and Ri are mathematically related (both derived from systolic and diastolic velocities), so when Pi is already in the model capturing the majority of the pulsatility effect, the model assigns a negative weight to Ri to fine-tune the fit. We do not interpret this as Ri truly decreasing with ICP, but rather as an adjustment within a correlated predictors set. ODH’s inclusion in the model, alongside ONSD, suggests that direct sonographic evidence of papilledema provides additive information about ICP, perhaps by indicating duration or chronicity of pressure elevation that might not be reflected by instantaneous ONSD alone. Interestingly, the bilateral mean Pi emerged as the single best predictor in univariable analysis of continuous ICP. Although this suggests that global cerebrovascular changes (captured by bilateral TCD) strongly reflect ICP variations, it does not negate the value of our multiparameter, side-specific strategy. A bilateral average may mask important lateral differences and lacks the input from ocular ultrasound parameters. Indeed, our combined affected side model outperformed any single metric by integrating both vascular and structural changes specific to the affected hemisphere. This indicates that a holistic approach is beneficial, even if one variable (such as bilateral Pi) is particularly powerful. These findings are consistent with those of Cardim et al. [30], who showed that combining several noninvasive ultrasound-derived indexes yields superior ICP predictions compared with any single parameter alone.

### Comparison with Previous Studies

Our findings reinforce and extend earlier work on noninvasive ICP estimation. Several investigators have already shown that TCD indexes possess a very high negative predictive value for ruling out intracranial hypertension [10, 11]. Most recently, the multicenter Invasive versus Noninvasive Measurement of Intracranial Pressure in Brain Injury Trial 2 (IMPRESSIT 2) confirmed that a single-side MCA-PI cutoff can safely exclude ICP ≥ 22 mm Hg, achieving a negative predictive value > 0.95 in patients with brain injury. We likewise observed that affected side ONSD or PI alone yields high AUCs and is well-suited to rapid triage. The advance of the present study lies in combining hemodynamic (PI, RI) and structural (ONSD, ODH, ONSD/ETD) markers and anchoring them to the hemisphere that bears the pathology. This side-specific, multiparameter model achieved a balanced sensitivity (80.8%) and specificity (75.8%) with an AUC ≈ 0.83, values comparable to the best single-parameter rules reported previously yet inherently more robust across diverse clinical scenarios [31, 32]. A pragmatic workflow therefore suggests itself: in the emergency department, a quick single-side TCD screen, as advocated by IMPRESSIT-2, can rapidly rule out dangerous ICP; patients with equivocal or positive screens can then undergo the more comprehensive, bilateral examination described here, especially in ICUs where continuous invasive monitoring is not feasible. By acknowledging these complementary strengths, we position our hemispheric multimodal ultrasound as a confirmatory—and potentially longitudinal—tool rather than a replacement for established rapid-screening algorithms.

### Affected Side Versus Global ICP Measurements

An inherent consideration in our study is that ICP itself was measured invasively either by intraparenchymal probes or EVDs. One might ask whether the superiority of affected side ultrasound was partly because, in many cases, the invasive monitor (especially intraparenchymal) was also located in the affected hemisphere. An intraventricular catheter, on the other hand, measures a more global pressure that could potentially underestimate focal pressure elevations in the affected side. Despite this, we observed the same trend of better affected side correlation even in patients monitored with EVDs, implying that substantial focal pressure increases do translate into higher global ICP. It remains possible that an EVD might “average out” some asymmetry, but our sample was too limited to conclusively compare EVD versus intraparenchymal subgroups. Future research with simultaneous bilateral ICP measurements could further elucidate how monitor type influences the relationship between regional ultrasound findings and overall ICP.

### Study Limitations

We acknowledge several limitations in our study. First, the sample size (41 patients) and number of measurements, although larger than many prior pilot studies, is still relatively modest for building predictive models—especially for complex models such as our multiparameter GLMM. This size constraint might limit the ultimate accuracy of our models and risks overfitting to our specific cohort. We mitigated this by using cross-validation and focusing on variables strongly supported by physiology and Lasso selection, but external validation in a larger independent cohort is essential. Second, although our measurements were prospectively collected, they were not strictly time-synchronized across patients (measurements were done at least twice daily as clinically indicated, rather than at fixed uniform intervals). This pragmatic design reflects real-world practice but could introduce bias if, for example, measurements were more likely to be taken during ICP crises in some patients. Third, the heterogeneity of patient pathologies (traumatic brain injury, stroke, tumors) means that our model captures a mix of mechanisms of intracranial hypertension; although this broadens applicability, it might reduce specificity for any single pathology. Dedicated models for more homogeneous patient groups might achieve higher precision. Fourth, as discussed, patients had either EVD or intraparenchymal ICP monitors, which themselves have different dynamics and response times; we did not stratify our analysis by monitor type. Fifth, about one quarter of our cohort (10 patients) had undergone primary decompressive craniectomy before ICU admission; although sensitivity analysis showed no material change in model performance after excluding these patients, decompressive craniectomy alters intracranial compliance and should be examined in larger datasets. Finally, the ultrasound techniques, particularly optic nerve measurements, have inherent interoperator variability. We attempted to minimize this by a standardized protocol (including color Doppler guidance to delineate the optic nerve sheath), yet ultrasound remains user dependent. In a busy ICU, obtaining consistent high-quality scans in every patient is challenging, and some patients (especially older individuals with poor temporal windows) may not be amenable to TCCD at all.

### Future Directions

Our study provides proof-of-concept for a hemispheric, multimodal ultrasound approach to noninvasive ICP monitoring. The next steps should include validating this strategy in larger, multicenter cohorts and investigating its prospective utility in clinical decision-making. For instance, can our model be used to trigger interventions or detect intracranial hypertension early, and would that improve patient outcomes? Additionally, real-time or automated implementations of this approach (potentially incorporating machine learning for continuous ICP prediction) could be explored [33–35]. Combining ultrasound with other noninvasive modalities (such as fundoscopy or EEG-based ICP indexes) might further enhance accuracy. As wearable or easy-to-apply ultrasound devices develop, a multiparameter model like ours could be integrated into noninvasive monitoring systems to provide continuous estimates of ICP at the bedside or even in prehospital settings.

## Conclusions

In summary, this dual-center prospective study confirms both the feasibility and the distinct clinical value of a side-specific, multimodal ultrasound strategy for noninvasive ICP monitoring. By integrating TCD and optic nerve sonography metrics from the lesion side, our multiparameter model achieved high accuracy and good robustness for continuous ICP estimation and for detecting intracranial hypertension, offering a measurable performance advantage over commonly used noninvasive approaches. These findings underscore the importance of capturing focal pathology and demonstrate that a multiparameter framework can furnish a more comprehensive basis for precise assessment. Although further validation in larger, more heterogeneous cohorts is still required, the present results lay a solid foundation for individualized, precision-oriented noninvasive ICP monitoring. Such an approach could, in appropriate scenarios, lessen reliance on invasive techniques in neurocritical care and facilitate earlier, more reliable detection of intracranial hypertension.

## Clinical Trial Registration

Trial registration: ChiCTR2400084669.
